# Demalonylation of DDX3 by Sirtuin 5 promotes antiviral innate immune responses

**DOI:** 10.7150/thno.52934

**Published:** 2021-05-24

**Authors:** Xingying He, Tianliang Li, Kewei Qin, Shiyuan Luo, Zhenjie Li, Qingqing Ji, Honghao Song, Huyang He, Hao Tang, Chaofeng Han, Hongjiao Li, Yan Luo

**Affiliations:** 1 Department of Anesthesiology, Changzheng Hospital, Naval Medical University, Shanghai, China.; 2 National Key Laboratory of Medical Immunology & Institute of Immunology, Naval Medical University, Shanghai, China.; 3 Central Laboratory and College of Otolaryngology Head and Neck Surgery, the Sixth Medical Center of Chinese PLA General Hospital, Beijing, China; 4 Department of Anesthesiology, Ruijin Hospital, Shanghai Jiaotong University School of Medicine, Shanghai, China.; 5 Department of Orthopedics, Changhai Hospital, Naval Medical University, Shanghai, China.; 6 Department of Histoembryology and Shanghai Key Laboratory of Cell Engineering, Naval Medical University, Shanghai, China.; 7 Department of Stomatology, Xinhua Hospital, Shanghai Jiaotong University School of Medicine, Shanghai, China.

**Keywords:** Sirtuin, DDX3, TBK1, IRF3, IFN, viral infection

## Abstract

**Rationale**: Hosts defend against viral infection by sensing viral pathogen-associated molecular patterns and activating antiviral innate immunity through TBK1-IRF3 signaling. However, the underlying molecular mechanism remains unclear.

**Methods**: SiRNAs targeting Sirt1-7 were transfected into macrophages to screen the antiviral function. *Sirt5* deficient mice or macrophages were subjected to viral infection to assess *in vivo* and *in vitro* function of Sirt5 by detecting cytokines, viral replicates and survival rate. Immunoprecipitation, WesternBlot and luciferase reporter assay were used to reveal molecular mechanism.

**Results**: In this study, we functionally screened seven Sirtuin family members, and found that Sirtuin5 (Sirt5) promotes antiviral signaling and responses. *Sirt5* deficiency leads to attenuated antiviral innate immunity* in vivo* and* in vitro* upon viral infection by decreasing TBK1-IRF3 activation and type I IFN production. Sirt5 overexpression increased antiviral innate immunity. Mechanism investigation revealed that Sirt5 interacts with DDX3 and demalonylates DDX3, which is critical for TBK1-IRF3 activation. Mutation of the demalonylation lysine sites (K66, K130, and K162) of DDX3 increased *ifnβ* transcription. Furthermore, the acetylation on lysine 118 of DDX3 positively regulated *ifnβ* transcription, whereas Sirt5 could not deacetylate this site.

**Conclusion**: Sirt5 promotes anti- RNA and DNA virus innate immune responses by increasing TBK1 signaling through demalonylating DDX3, which identifies a novel regulatory pathway of antiviral innate immune response.

## Introduction

The innate immune system provides the first line of defense against viral infections, such as COVID-19 and influenza, through pattern-recognition receptor (PRR)-initiated antiviral responses 1-3. PRRs detect various conserved pathogen-associated molecular patterns (PAMPs), including viral double-stranded RNA (dsRNA), single-stranded RNA (ssRNA), and DNA 1, 2. Toll-like receptor 3 (TLR3) in the endosome detects viral dsRNA and triggers TLR-domain-containing adapter-inducing interferon-β (TRIF)-dependent nuclear factor-κB (NF-κB) and interferon regulatory factor 3 (IRF3) activation. TLR7 and TLR8 in the endosome recognize viral single-stranded RNA (ssRNA) and induce myeloid differentiation primary response 88 (MyD88)-dependent NF-kB activation. However, RNA from viruses is mostly in the cytoplasm. Retinoic acid-inducible gene 1 (RIG-I) or melanoma differentiation-associated protein 5 (MDA5) detect and activate mitochondrial antiviral-signaling protein (MAVS)-mediated IRF3/7 and NF-kB signaling pathways 4, 5. Cytoplasmic viral DNA is recognized by gamma-interferon-inducible protein-16 (IFI16) and cyclic GMP-AMP (cGAMP) synthase (cGAS) that activate the IRF3/7 and NF-κB signal pathways and induce the expression of type I interferon (IFN-I), interferon-stimulated genes (ISGs), and proinflammatory cytokines through stimulator of IFN genes (STING) 6,7. Innate immune responses are orchestrated by positive and negative regulation to limit viral infection and avoid tissue damage. Posttranslational modifications (PTMs) of PRRs and downstream signaling molecules, including phosphorylation, ubiquitination, methylation, and acetylation, are critical for antiviral immune responses and disease pathogenesis 8-10. Metabolic regulation of innate immunity, such as glycolysis is required for anti-bacterial inflammation while it inhibits antiviral infection, needs further investigation 11, 12. Moreover, the signal transduction mechanisms of both host defense and viral immune escape also remain to be further explored.

RNA helicases contain many members, including viral RNA sensor RIG-I (DDX58). They are highly conserved enzymes that utilize the energy derived from NTP hydrolysis to modulate the structure of RNA. The DEAD motif (for the sequence Asp-Glu-Ala-Asp) proteins can unwind double stranded RNA structures and regulate RNA protein interactions via ATPase enzyme activity. DDX3 subfamily contain two members. DDX3X (so called DDX3) protein is expressed in all tissues and higher in females than in males. DDX3Y protein expression is restricted to the male germ cells with the specific role in spermatogenesis. DDX3 can regulate RNA transcription, pre-mRNA splicing, RNA export and translation and also regulate transcriptor such as p53, β-catenin activation. However, whether it functions as an oncogene or a tumor suppressor was controversial, which may result from the different protein modification. DDX3 shifts from the nucleus to the cytoplasm by interacting with exportin 1, which will regulate HCV and HIV replicate. DDX3 also interacts with influenza A virus NS1 and NP proteins and exerts antiviral function through regulation of stress granule formation and can directly impinge on IFN-β promoters to regulate their transcription as reviewed recently 13, 14. DDX3 recently was found to interact with NLRP3 to drive inflammasome activation 15. However, the activation mechanism of DDX3, such as protein modification, remains to be elusive.

Silent information regulator 2 (Sir2) proteins (sirtuins) are nicotinamide adenine dinucleotide (NAD) -dependent deacetylases that regulate important biological processes, such as survival, aging, inflammatory response, stress response, metabolism, and necroptosis 16-18. Sirtuin 5 (Sirt5), which primarily resides in the mitochondria, regulates metabolism in physiological and pathophysiological conditions by its desuccinylase, demalonylase, and deglutarylase activity 19, 20. Sirt5 regulates glycolysis, tricarboxylic acid (TCA) cycle, electron transport chain, urea cycle, fatty acid oxidation, ketone body formation, and reactive oxygen species (ROS) detoxification in mitochondria 19-26. Moreover, the distribution of Sirt5 in extra-mitochondrial compartments suggests a potential role for Sirt5 in cytosolic and nuclear processes 20. For example, Sirt5 contributes to tumorigenesis and migration; it acts as a tumor promoter in some cases and tumor suppressor in others 19, 27. We previously reported that cytoplasmic Sirt5 enhanced inflammatory responses in macrophages and endotoxin-tolerant macrophages by blocking the deacetylation of p65 by Sirt2 28. However, the biological role of Sirt5 in antiviral innate immunity remains elusive.

In this study, we demonstrated that Sirt5 promotes antiviral innate immune response *in vivo* and *in vitro*. We found that Sirt5 interacts with and demalonylates DDX3 to increase the activation of TBK1 and IFN production. Malonylating site mutants of DDX3 caused increased *ifnβ* transcription. Our data reveal a novel mechanism through which Sirt5 promotes antiviral innate immune response in both macrophages and epithelial cells by demalonylating DDX3.

## Materials and methods

### Mice

B6;129-Sirt5^tm1Fwa^/J (*Sirt5^-/-^*) mice were purchased from the Jackson Laboratory. C57BL/6J mice (6-8 weeks old) were obtained from Joint Ventures Sipper BK Experimental Animal Company (Shanghai, China). *Sirt5^+/+^*, *Sirt5^+/-^*, and *Sirt5*^-/-^ homozygote littermates were derived by mating the* Sirt5^+/-^* heterozygote mice with each other 28. All mice were bred in specific pathogen-free conditions. All animal experiments were performed in accordance with the National Institute of Health Guide for the Care and Use of Laboratory Animals with approval from the Scientific Investigation Board of the Second Military Medical University, Shanghai, China.

### Reagents

Antibodies against RIG-I, phospho-TBK1, TBK1, MAVS, phospho-IRF3, IRF3, phospho-c-Jun, c-Jun, Sirt5, actin, myc-tag, and the HRP-conjugated secondary antibody were purchased from Cell Signaling Technology. Antibodies against DDX3X were from Abcam. Antibodies against V5-tag (HRP-conjugated) were purchased from Medical & Biological Laboratories. Anti-V5 Agarose Affinity Gel antibody used in immunoprecipitation was from Sigma-Aldrich. Antibodies against malonyl-lysine, succinyl-lysine, and glutaryl-lysine were from PTM BioLab. Poly(I:C) (HMW) / LyoVec™, Poly(I:C) (LMW) / LyoVec™, ssRNA40/LyoVec™ and CyQUANT™ LDH were from InvivoGen.

### Cell culture

Murine macrophage cell line (RAW264.7), human embryonic kidney cell line (HEK293T), and human lung epithelial adenocarcinoma cell line (A549) were obtained from American Type Culture Collection. Bone marrow-derived macrophages (BMDMs), peritoneal macrophages, dendritic cells and bone marrow derived stroma cells were prepared as described previously 28, 29. All cells were cultured in Dulbecco's modified Eagle's medium (DMEM) (Gibco) with 10% fetal bovine serum (Gibco) in a 5% CO_2_ atmosphere at 37 °C.

### Plasmid constructs and transfection

Recombinant plasmids encoding Sirt5, DDX3X, and DDX3X point mutants were constructed from cDNA of RAW264.7 cells using PCR-based amplification, and then subcloned into the pcDNA3.1 eukaryotic expression vector as described previously 29,30. The recombinant plasmids were transfected into cells using jetPEI (Polyplus Transfection) following the manufacturer's instructions.

### RNA interference

Small interfering RNAs (siRNAs) were transfected into cells using RNAiMAX Transfection Reagent (Thermo Fisher) according to the manufacturer's instructions.

### Luciferase reporter assay

HEK293T cells were plated in 24-well plates, cultured for 12 h, and then transfected with IFN-β reporter plasmid with the indicated expression plasmids, with Renilla luciferase plasmid serving as an internal control. After 24 h of transfection, the cells were lysed, and the lysates were collected to measure luciferase activity using the Dual-Luciferase Reporter Assay System (Promega) according to the manufacturer's instructions 28, 29.

### RNA quantification

Total RNA was extracted using RNA extraction kit (Fastagen) or TRIzol reagent (Transgen). RNA was reverse-transcribed using reverse transcription kit (TOYOBO) and analyzed using SYBR quantitative real-time-PCR kit (Takara) with a LightCycler (Roche). All data were normalized to *Gapdh* expression 28, 29.

### Enzyme-linked immunosorbent assay (ELISA)

Serum from mice or cell culture supernatants were diluted and analyzed using IFN-β (PBL Biomedical Laboratories), TNF-α (R&D Systems) and IL-6 (R&D Systems) ELISA kits according to the manufacturer's instructions 28, 29.

### Immunoprecipitation and immunoblotting

Cells were plated in 6-well plates, cultured for 12 h, and transfected with the expression plasmids. After 24 h, the cells were lysed using lysis buffer (Cell Signaling Technology) containing 1% protease inhibitor cocktail (Calbiochem) and then centrifuged. Protein concentrations in the supernatant were measured using BCA assay (Thermo fisher). The cell lysate was incubated with Agarose Affinity Gel antibody for 2 h for immunoprecipitation. Immunoprecipitation and immunoblotting were performed as described previously 28, 29.

### Viral infection *in vitro* and *in vivo*

Mice (6-8 weeks old) were injected intraperitoneally with Vesicular stomatitis virus (VSV, Indiana Strain) at the indicated doses (plaque-forming units per gram body weight, PFU/g) and times. The concentration of cytokines in the serum was measured by ELISA. The mRNA expression of cytokines was measured by Q-PCR. Cells were infected with VSV (MOI, 1), Sendai virus (SeV, Japan Stain, MOI, 1), or Herpes simplex virus type 1 (HSV-1, Kos Strain, MOI, 10) for the indicated durations as previously reported 29. Cytokine production was analyzed by ELISA and mRNA expression was measured by Q-PCR 29, 31.

### Statistical analysis

Data are presented as mean ± standard deviation (SD). Comparisons between two groups were analyzed using Student's *t*-test. Survival curves were constructed with Kaplan-Meier estimates, and survival rates were analyzed using the generalized Wilcoxon's test. P < 0.05 was considered statistically significant.

## Results

### Functional Screening of Sirtuin family identifies Sirt5 as a positive regulator of IFN-β

**Figure 1:**
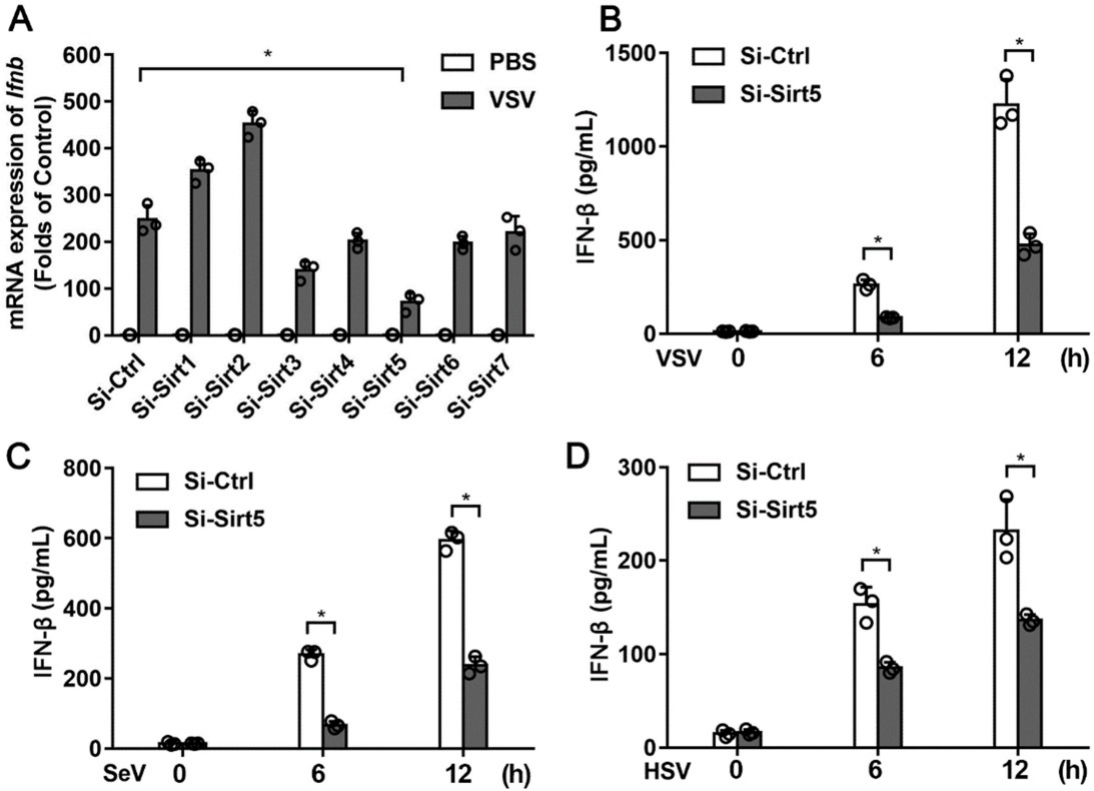
Identification of Sirt5 as a positive regulator of type I interferon expression. **(A)** Q-PCR analysis of *Ifnβ* mRNA in BMDMs transfected with siRNA specific for Sirt1-7 for 48 h and then infected with VSV for 6 h. The results are normalized to *Gapdh* expression. **(B-D)** ELISA for IFN-β in supernatant of BMDMs transfected with Sirt5-siRNA for 48 h and then infected with VSV **(B)**, SeV **(C),** and HSV **(D)** for the indicated durations. Data are presented as mean ± standard deviation (SD) of three independent experiments. (* P < 0.05).

To examine the functional role of Sirtuin family members in antiviral innate immunity, we knocked down the expression of Sirt1-7 in BMDMs using siRNA (Figure S1A). The protein of expression of Sirt5 was also efficiently decreased (Figure S1B). Knocking-down of Sirt5 expression significantly decreased VSV-induced IFN-β mRNA expression (Figure 1A), which indicates that Sirt5 may play a positive role in antiviral innate immune response. Furthermore, the productions of IFN-β and inflammatory cytokines in Sirt5-knockdown BMDMs following VSV infection were significantly reduced compared to that in the control cells (Figure 1B, Figure S2). Consistent with these results, RNA virus (SeV)- or DNA virus (HSV)-induced IFN-β production was also significantly decreased in the Sirt5-knockdown cells (Figure 1C-D). These results indicate that Sirt5 increases viral infection-induced IFN-β production.

### *Sirt5* deficiency impairs the antiviral immune response *in vivo and in vitro*

**Figure 2:**
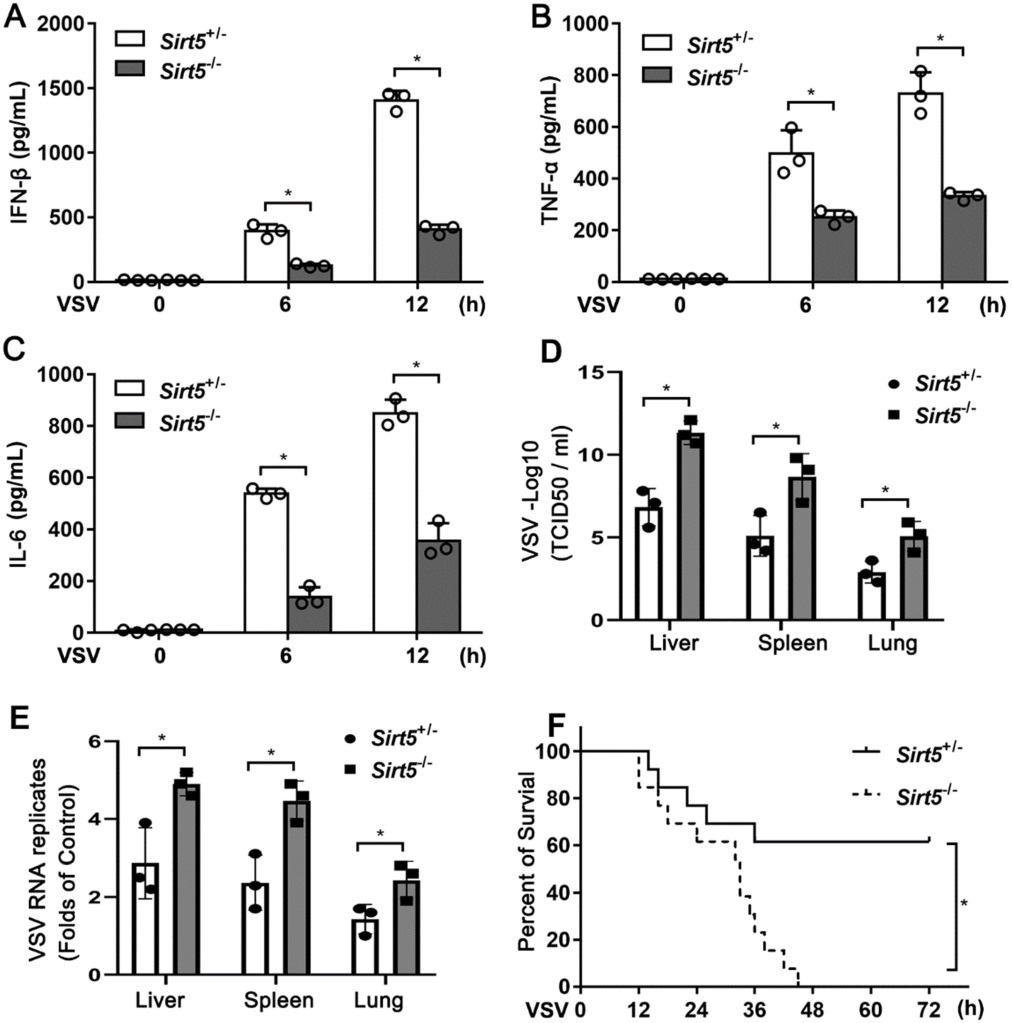
Deficiency of Sirt5 impairs antiviral innate immune response *in vivo*.** (A-C)** ELISA for IFN-β (A), TNF-α **(B),** and IL-6 **(C)** in serum of *Sirt5*^+/-^ and *Sirt5*^-/-^ mice (n = 3) intraperitoneally injected with VSV (10^8^ plaque-forming units per gram of body weight) for indicated durations.** (D)** VSV loads in the liver, lungs, and spleens of *Sirt5*^+/-^ and *Sirt5*^-/-^ mice (n = 3) 12 h after intraperitoneal injection with VSV (10^8^ PFU/g) **(E)** Real-time PCR analysis of VSV RNA levels in livers, spleens, and lungs of *Sirt5*^+/-^ and *Sirt5*^-/-^ mice (n = 3) 12 h after intraperitoneal injection with VSV (10^8^ PFU/g) **(F)** Survival of *Sirt5*^+/-^ mice and *Sirt5*^-/-^ mice after intraperitoneal injection of VSV (4 × 10^8^ PFU/g) (n = 10) Data are presented as mean ± SD of three independent experiments. (*P < 0.05).

To investigate the pathological function of Sirt5 *in vivo*, we infected *Sirt5*^-/-^ mice and *Sirt5*^+/-^ littermates with VSV. The productions of IFN-β and proinflammatory cytokines (TNF-α and IL-6) in the serum of *Sirt5*^-/-^ mice were significantly lower compared to that in the control mice (Figure 2A-C). The viral load in the liver, lung, and spleen of *Sirt5*^-/-^ mice was significantly higher than that in control mice (Figure 2D). VSV RNA levels in the infected organs of *Sirt5^-/-^* mice were higher than that in the organs of control mice (Figure 2E). Moreover, *Sirt5*^-/-^ mice were more susceptible to VSV infection than *Sirt5*^+/-^ littermates (Figure 2F). Together, these results demonstrate that Sirt5 promotes antiviral innate immune response *in vivo*.

**Figure 3:**
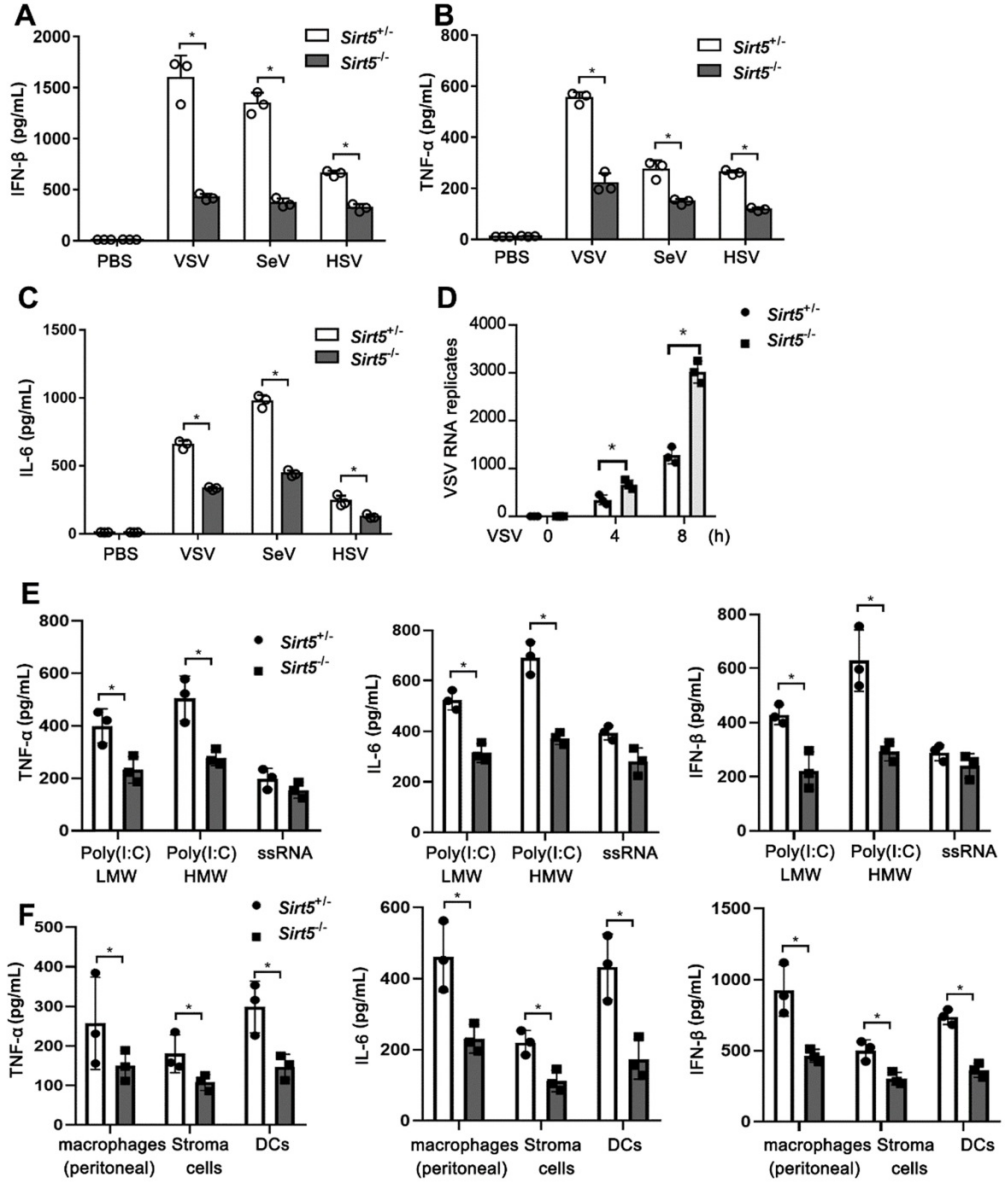
*Sirt5* deficiency impairs antiviral innate immune responses *in vitro*. **(A-C)** ELISA for IFN-β, TNF-α, and IL-6 levels in supernatant of *Sirt5*^+/-^ and *Sirt5*^-/-^BMDMs infected with VSV, SeV, or HSV for 12 h. **(D)** Real-time PCR analysis of VSV RNA levels in *Sirt5*^+/-^ and *Sirt5*^-/-^ BMDMs infected with VSV for the indicated durations. **(E)** ELISA for IFN-β, TNF-α, and IL-6 levels in supernatant of *Sirt5*^+/-^ and *Sirt5*^-/-^ BMDMs stimulated with Poly(I:C) (HMW) / LyoVec™ (1 μg/mL), Poly(I:C) (LMW) / LyoVec™ (1 μg/mL ), ssRNA40/LyoVec™ (1 μg/mL ) for 12 h. **(F)** ELISA for IFN-β, TNF-α, and IL-6 levels in supernatant of *Sirt5*^+/-^ and *Sirt5*^-/-^ peritoneal macrophages, bone marrow derived stroma cells and DCs infected with VSV for 12 h. Data are presented as mean ± SD of three independent experiments. (*P < 0.05).

Next, we evaluated the function of Sirt5 in antiviral immune responses *in vitro*. We infected *Sirt5*^-/-^ and *Sirt5*^+/-^ BMDMs with VSV, SeV, and HSV. The productions of IFN-β and proinflammatory cytokines (TNF-α and IL-6) in *Sirt5*^-/-^ BMDMs were significantly decreased compared to that in *Sirt5^+/-^* control BMDMs (Figure 3A-C). However, the viral loads of VSV were significantly higher in *Sirt5^-/-^*BMDMs compared to that in *Sirt5^+/-^* control BMDMs (Figure 3D). We previously reported that Sirt5 increased NF-kB activation by increasing acetylation of p65 via competing with Sirt2, which promoted TLR4-induced inflammatory cytokines 28. To explore function of Sirt5 on anti-viral innate immunity in the kinetic time course, we tested virus induced the IFN-β production and LDH release at 0, 12, 24 and 48 h in *Sirt5^+/-^* and *Sirt5^-/-^* BMDMs. We found that IFN-β production was increased 12 and 24 h after viral infection (Figure S3A). The IFN-β production was decreased 48 h after viral infection compared to 12 h and 24 h, which may result from cell death and protease release. The LDH release was increased gradually with time course, typically over 50% cell died 48 h after infection (Figure S3B). We speculated that impaired IFN-β production in *Sirt5^-/-^* BMDMs lead to uncontrolled viral proliferation and increased cell death. Accordingly, *Sirt5* deficiency decreased IFN-β production and increased LDH release (Figure S3A-B).

To investigate the function of Sirt5 on interferons transcription, we tested the mRNA of IFN-α and IFN-γ in *Sirt5^+/-^* and *Sirt5^-/-^* BMDMs. *Sirt5* deficiency decreased significantly the mRNA of expression IFNα, while did not deceased significantly IFN-γ expression significantly (Figure S3B), which may be resulted from different signal pathway IFN-γ (STAT1/2) and IFN-α/β (IRF3/7) transcription.

To further illustrate the function of Sirt5 in various PRRs, we tested the function of Sirt5 on proinflammatory cytokines and IFN-β production upon short poly I:C, long poly I:C, and ssRNA stimulation and found that *Sirt5* deficiency also decreased Poly(I:C) (Low Molecular Weight, LMW) and Poly(I:C) (High Molecular Weight, HMW) induced inflammatory cytokines and IFN-β in macrophages, which are sensed by RIG-I/MDA5 dependent pathway (Figure 3E). However, *Sirt5* deficiency only decreased ssRNA40/Lyo Vec™ -induced inflammatory cytokines and IFN-β in macrophages without statistical significance, which is sensed by TLR7 dependent pathway (Figure 3E). To further explore the role of Sirt5 in various cell types, we tested the function of Sirt5 on proinflammatory cytokines and IFN-β production in peritoneal macrophages, bone marrow stroma cells and DCs upon on both ssRNA40/Lyo Vec™ and VSV stimulation, and found that *Sirt5* deficiency also decreased VSV induced inflammatory cytokines and IFN-β in these cells (Figure 3F), while not ssRNA40/Lyo Vec™ induced inflammatory cytokines and IFN-β (Figure S4).

Above data suggest that Sirt5 increased cytoplasmic RNA and DNA sensing receptors induced anti-viral responses, which draws a conclusion that Sirt5 promotes antiviral innate immune response *in vivo* and* in vitro*.

### *Sirt5* deficiency impairs antiviral signal transduction

**Figure 4:**
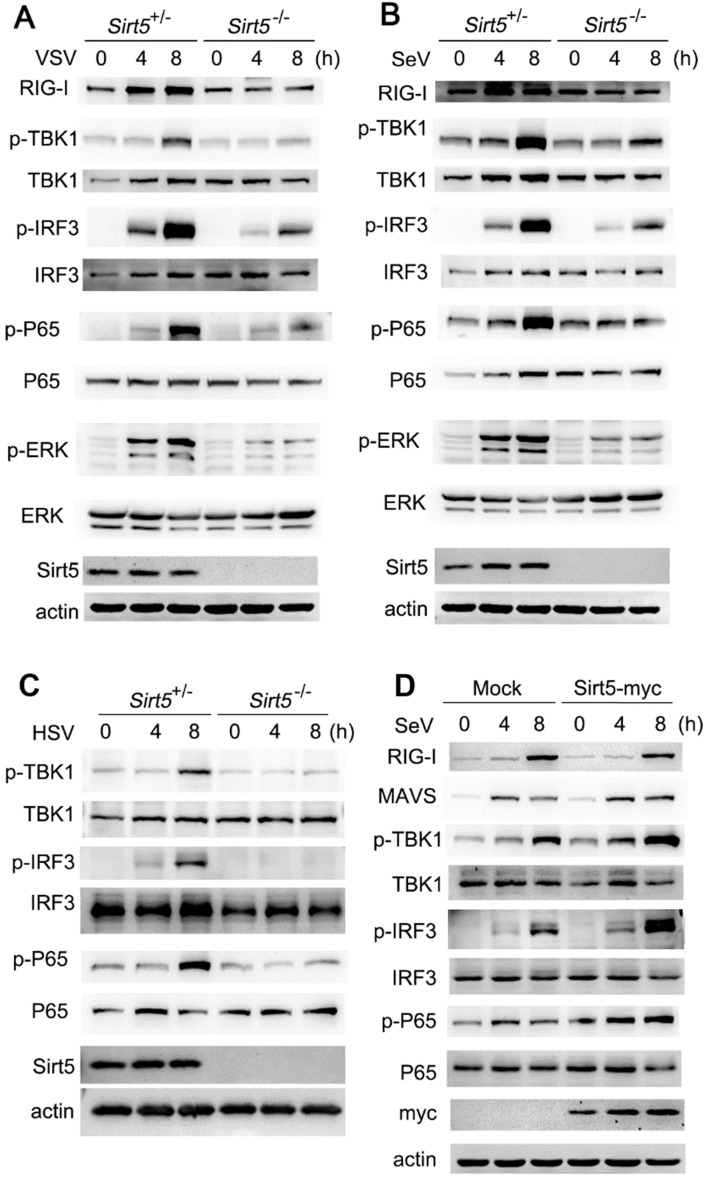
Sirt5 promotes antiviral innate immune signaling pathways. **(A-C)** Immunoblot analysis of *Sirt5*^+/-^ and *Sirt5*^-/-^BMDMs infected with VSV, SeV, or HSV for the indicated durations. **(D)** Immunoblot analysis of A549 cells transfected with Sirt5 for 48 h and then infected with SeV for indicated durations. Data are representative of three independent experiments.

To determine the mechanism through which Sirt5 promotes antiviral immunity, we analyzed the activation of signaling pathways using western blotting. We found that the activation of TBK1, IRF3, and MAPK pathways were decreased in *Sirt5*^-/-^ BMDMs following VSV infection compared to the *Sirt5*^+/-^ control BMDMs (Figure 4A), which indicates that Sirt5 may function on the upstream of viral sensing signaling. Accordingly, the activation of the TBK1-IRF3 signaling pathway was also decreased in *Sirt5*^-/-^ BMDMs upon SeV or HSV infection (Figure 4B, C and Figure S5). In addition, Sirt5 overexpression increased the phosphorylation of TBK1 and IRF3 in A549 cells upon SeV infection. Taking into account that *Sirt5* deficiency in peritoneal macrophages, bone marrow stroma cells and DCs also decreased anti-viral responses, the positive role of Sirt5 overexpression further indicates that Sirt5 has positive role in anti-viral responses and signal transduction in various cell types. These results suggest that Sirt5 promotes antiviral immune responses by promoting the activation of TBK1-IRF3 signaling pathway during VSV, SeV, and HSV infection.

### Sirt5 interacts with DDX3

To further understand the mechanism through which Sirt5 promotes antiviral innate immune response, we established RAW264.7 cells that stably overexpressed Sirt5 and performed co-immunoprecipitation and mass spectrometric assays using mock RAW264.7 cells as control. By analyzing Sirt5-interacting molecules, we identified DDX3, a member of DEAD (Asp-Glu-Ala-Asp)-box containing ATPase-dependent RNA helicase family, as a potential Sirt5-interacting molecule (Figure S6 and Table S1).

DDX3 has been found in various organisms from yeast to human and shuttles between the cytoplasm and nucleus 14. It has been reported that DDX3 promotes IFN-β production through interaction with TBK1/IKKε, IKKα, and IPS1 32-35. DDX3 also binds to the NF-κB subunit p65 and inhibits its activation 36. However, the mechanism of activation of DDX3 during antiviral responses remains unknown.

**Figure 5:**
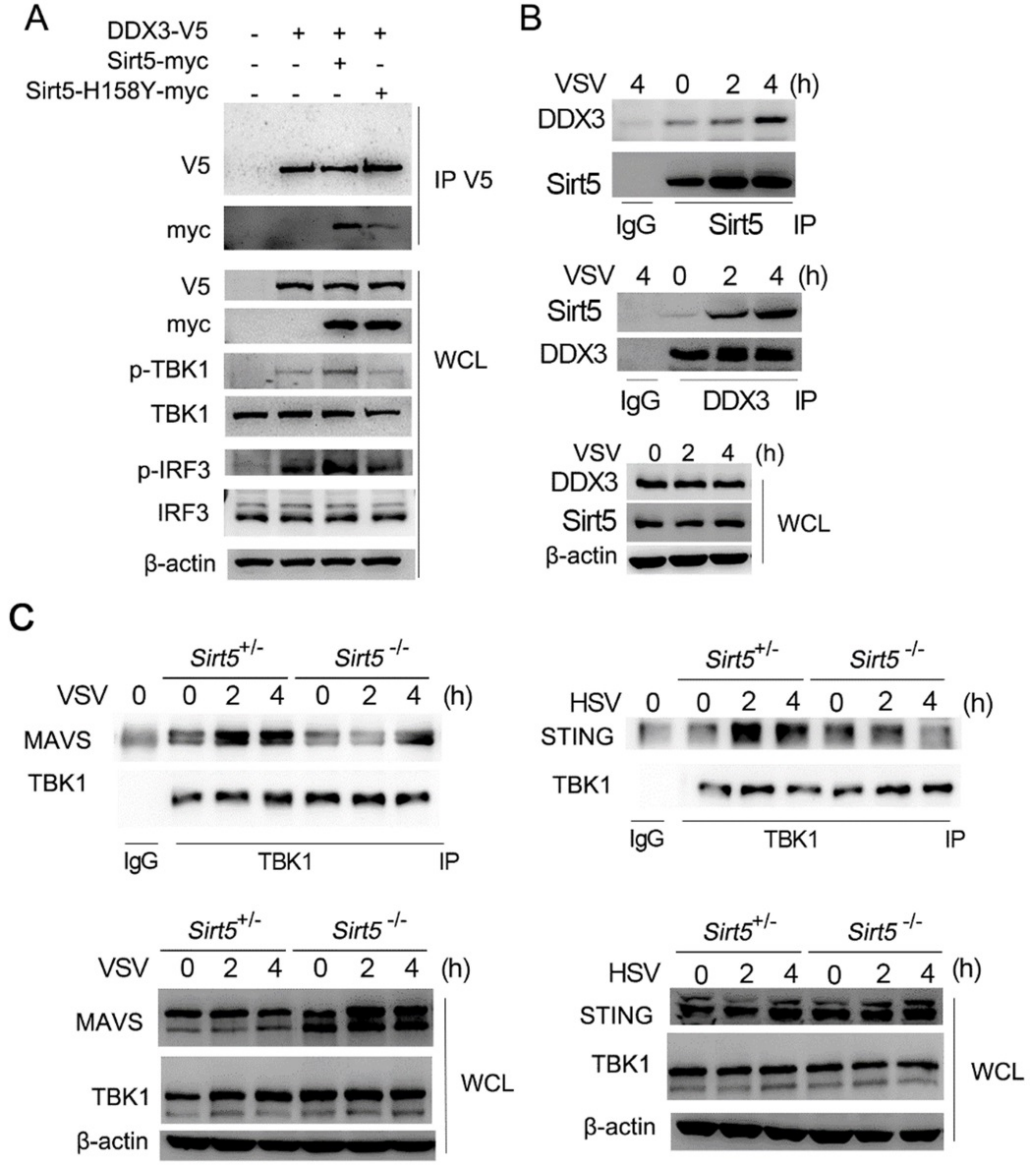
Sirt5 interacts with DDX3 in an enzyme activity-dependent manner. **(A)** Immunoblot analysis of co-immunoprecipitation and cell lysate from HEK293T cells transfected with DDX3, Sirt5, and Sirt5-H158Y with indicated antibodies. **(B, C)** Immunoblot analysis of co-immunoprecipitation and cell lysate from *Sirt5*^+/-^ and *Sirt5*^-/-^ BMDMs with indicated antibodies. Data are representative of three independent experiments.

We then further evaluated the interaction of DDX3 with Sirt5. Overexpressed DDX3 was found to interact with overexpressed Sirt5 in HEK293T cells (Figure 5A). DDX3 overexpression alone increased the activation of TBK1 and IRF3 in HEK293T cells, which was further enhanced by the overexpression of Sirt5-WT (Figure 5A). A kinase dead mutant, Sirt5-H158Y 20, 23, 37, interacted with DDX3 weakly and barely promoted the activation of TBK1 and IRF3 compared to Sirt5-WT (Figure 5A). The interaction between endogenously expressed Sirt5 and DDX3 was also detected in BMDMs following viral infection (Figure 5B). Further, we found that the formation of the signal complex between TBK1 with MAVS and STING was decreased in *Sirt5^-/-^* BMDMs compared to control cells following VSV and HSV infection (Figure 5C). Together, these results indicate that Sirt5 promotes TBK1-IRF3 activation by interacting with DDX3, and in a kinase activity-dependent manner.

### Sirt5 promotes antiviral innate immune response via DDX3

**Figure 6:**
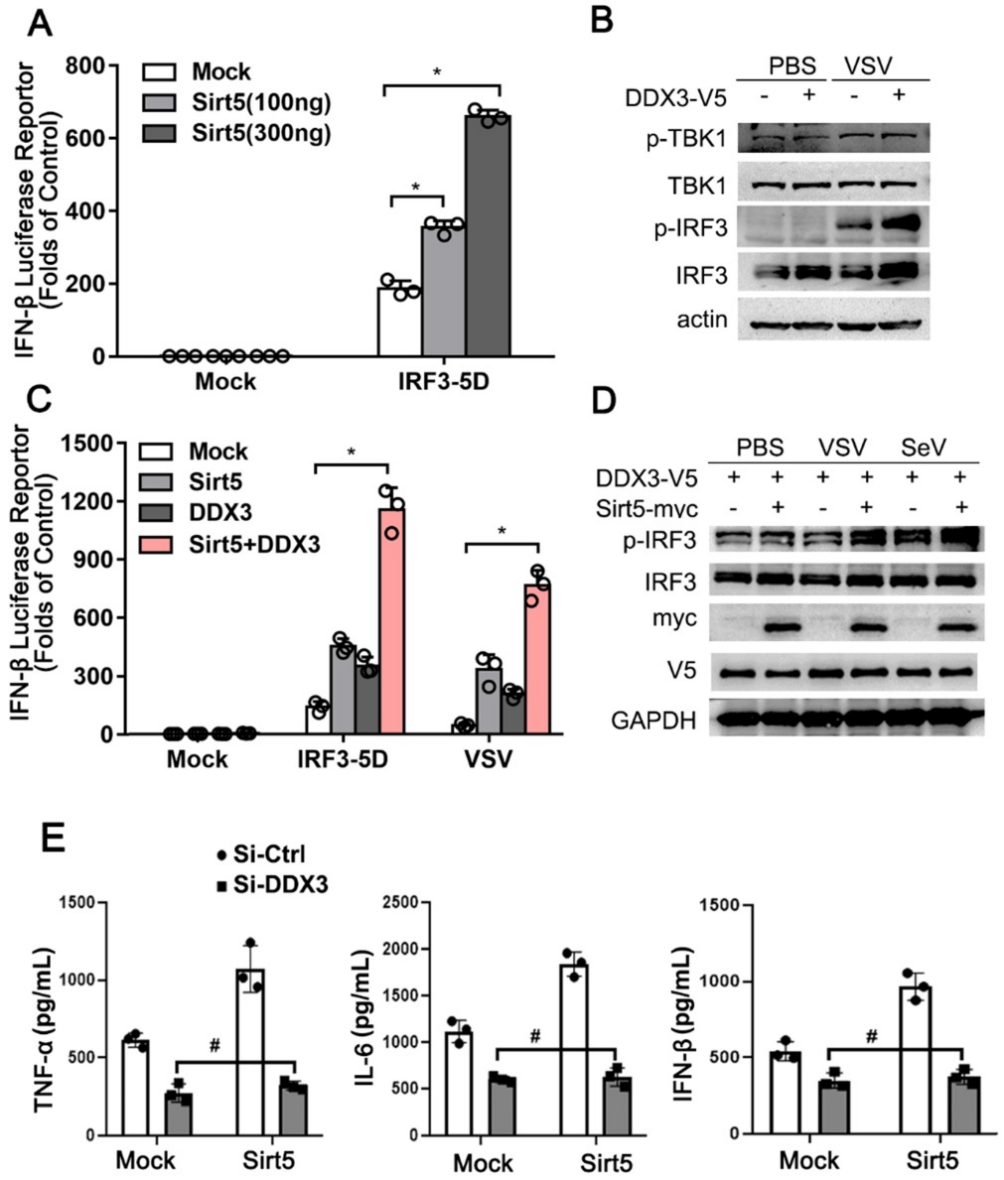
Sirt5 promotes DDX3-mediated IFN-β production. **(A)** Luciferase activity in HEK293T cells transfected with IFN-β luciferase reporter, constitutively active IRF3 (IRF3-5D), and Sirt5. The results are expressed relative to Renilla luciferase activity. **(B)** Immunoblot analysis of A549 cells transfected with DDX3 for 48 h and then infected with VSV for 8 h. **(C)** Luciferase activity in A549 cells transfected with IFN-β luciferase reporter, DDX3, and Sirt5 with IRF3-5D, or infected with VSV for 8 h. The results are expressed relative to Renilla luciferase activity. **(D)** Immunoblot analysis of A549 cells transfected with DDX3 and Sirt5 for 48 h and then infected with VSV or SeV for 8 h. **(E)** ELISA for IFN-β, TNF-α, and IL-6 levels in supernatant of RAW264.7 cells stably overexpressed with Sirt5 or empty vector transfected with Si-RNA targeting DDX3 or scramble control and then infected with VSV for 12 h. Data are presented as mean ± SD of three independent experiments or representative of three independent experiments. (* *P* < 0.05, # *P* > 0.05).

We further explored the molecular mechanism of the positive role of Sirt5 and DDX3 in antiviral innate immune response. Overexpressed Sirt5 dose-dependently promoted IRF3-mediated IFN luciferase reporter activity in HEK293T (Figure 6A). In line with these results, overexpressed DDX3 alone promoted the activation of TBK1 and IRF3 upon VSV infection in A549 cells (Figure 6B). In addition, co-overexpression of Sirt5 and DDX3 alone or together increased IRF3-mediated or VSV-induced *ifnβ* transcription in A549 cells (Figure 6C). Furthermore, the phosphorylation of IRF3 was also increased following co-overexpression of Sirt5 and DDX3 upon VSV or SeV infection in A549 cells (Figure 6D). Knocking-down of the endogenous expression of DDX3 in stably overexpressed Sirt5 RAW264.7 cells, abolished the positive function of Sirt5 on IFN-β and inflammatory cytokines (Figure 6E), which further strengthens that the positive function of Sirt5 in anti-viral innate immunity is dependence on DDX3. These results indicate that Sirt5 promotes DDX3-mediated antiviral responses.

### Demalonylation of DDX3 by Sirt5 increases *ifnβ* transcription

**Figure 7:**
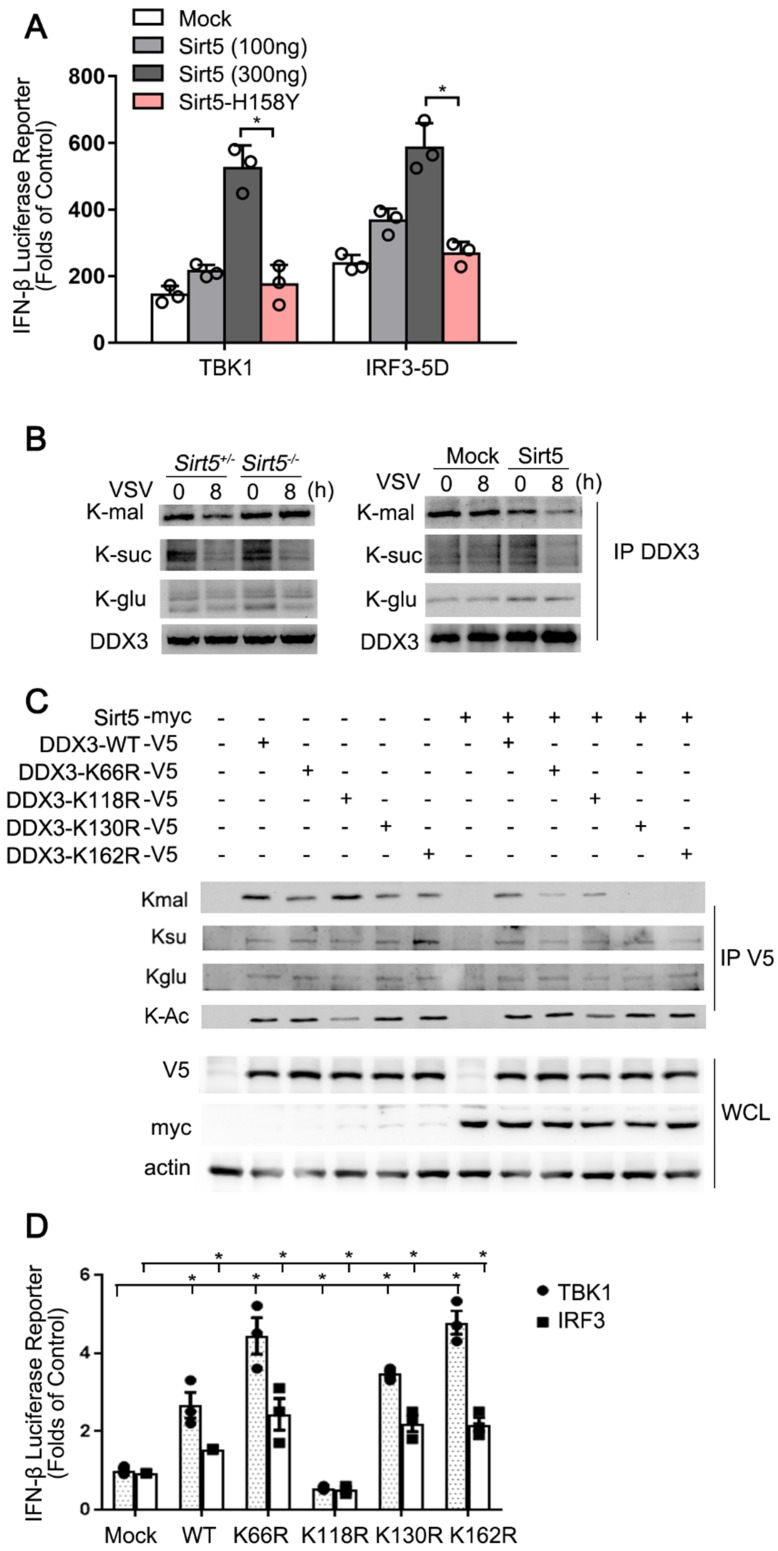
Sirt5 demalonylates DDX3. **(A)** Luciferase activity in HEK293T cells transfected with IFN-β luciferase reporter, TBK1, and IRF3-5D, together with empty vector (Mock), Sirt5 or Sirt5-H158Y (kinase dead mutants). Results are expressed relative to Renilla luciferase activity. **(B)** Immunoblot analysis of immunoprecipitated DDX3 from cell lysate of *Sirt5*^+/-^ and *Sirt5*^-/-^ BMDMs (left), or Sirt5 stably overexpressed or empty vector RAW264.7 cells (right) infected with VSV for indicated time and detected with indicated antibodies. **(C)** Co-immunoprecipitation and immunoblot analysis of overexpressed DDX3 with or without Sirt5 using the indicated antibodies. **(D)** Luciferase activity in HEK293T cells transfected with IFN-β luciferase reporter, TBK1, and IRF3-5D, together with empty vector (Mock), and DDX3-WT and mutant. The results are expressed relative to the Renilla luciferase activity. Data are presented as mean ± SD of three independent experiments or representative of three independent experiments. (* *P* < 0.05).

Given that Sirt5 has robust lysine desuccinylase, demalonylase, and deglutarylase activities 8, 20, 38, we tested whether the positive role of Sirt5 in antiviral immune response was enzyme activity-dependent. Overexpression of Sirt5 promoted TBK1 and IRF3-mediated *ifnβ* transcription. However, the overexpression of a kinase dead mutant of Sirt5 did not (Figure 7A). These results indicate that Sirt5 upregulates IFN-β transcription in a kinase activity-dependent manner.

Next, we first found that Sirt5 overexpression in RAW264.7 cells decreased while *Sirt5* deficiency increased the malonylation of DDX3 in BMDMs (Figure 7B). Then we overexpressed Sirt5 and DDX3 in HEK293T cells to examine whether Sirt5 affects PTM of DDX3. Overexpression of Sirt5 decreased the level of malonylation of DDX3-WT (Figure 7B). In contrast, the succinylation, glutarylation and acylation of DDX3 were not altered following overexpression of Sirt5 (Figure 7C), possibly due to the low level of these two modifications. Following a thorough search of several databases, we identified K66, K118, K130, and K162 as potential malonylation sites in DDX3 20, 39. We mutated K66, K118, K130, and K162 to arginine (R) and transfected HEK293T cells with DDX3-WT or these mutants individually. Malonylation of DDX3 was attenuated by K66R, K130R and K162 mutation, but not by the K118R mutation (Figure 7C). The acylation of DDX3 was decreased in the K118R mutant, while Sirt5 overexpression had no effect on the acylation of DDX3, possibly due to the low deacetylase enzyme activity of Sirt5. Next, we investigated whether demalonylation of DDX3 affects IFNβ transcription. Overexpression of DDX3-K118R significantly decreased TBK1 and IRF3-mediated *Ifnβ* transcription in HEK293 cells, however K66R, K130R, and K162R mutants of DDX3 significantly increased TBK1 and IRF3-mediated *Ifnβ* transcription in HEK293 cells (Figure 7D), which indicates that the demalonylation of DDX3 by Sirt5 promotes *Ifnβ* transcription. Taken together, these data suggest that Sirt5-mediated lysine demalonylation of DDX3 increases* Ifnβ* transcription and subsequently the antiviral innate immune response.

## Discussion

In this study, we report the positive role of Sirt5 in antiviral innate immune response following functional analysis of seven Sirtuin family members. *Sirt5* deficiency significantly decreases the production of IFN-β and inflammatory cytokines *in vivo* and in *vitro* following viral infection. *Sirt5*^-/-^ mice are more susceptible to viral infection accompanied with more viral load compared to control mice. Further analyses showed that Sirt5 promotes antiviral response by demalonylating K66, K130, and K162 of DDX3. The malonylation site mutants of DDX3 increased *ifnβ* transcription, which provides novel molecular mechanism for antiviral innate immune response.

PTMs regulate cellular behaviors during pathological and physiological processes through their ability to alter the activity, stability, compartmentalization, trafficking, and physical interaction of key molecules. The various types of PTMs include phosphorylation, ubiquitination, methylation, acetylation and other unconventional acylation, including succinylation, malonylation, glutarylation, propionylation, butyrylation, and crotonylation 9,40. Numerous kinases/phosphatases, ubiquitinases/deubiquitinases, methylases/demethylases, and acylases/deacylases regulate the reversible PTMs. However, the substrates of these new PTMs remain insufficiently explored, which greatly hinders progress in interpreting their regulatory mechanisms and cellular functions.

Sirtuins (Sirt1-7) are NAD^+^-dependent Lysine deacetylases that regulate diverse biological processes, including aging, metabolism, tumorigenesis, and inflammation 16, 41. Sirt5, which resides mostly in the mitochondria and partly in the cytoplasm, is a deacetylase that removes lysine succinylation, malonylation, and glutarylation from targeted proteins 20, 25, 38. Recently, proteomic analyses of protein acylation have identified the sites of protein succinylation, glutarylation, and malonylation in the cytosolic and nuclear fractions and their regulation by Sirt5 20, 38. However, the biological role of Sirt5 and acylation, in pathological states such as during viral infection, remains elusive. Our study shows that Sirt5 promotes antiviral innate immune response in a demalonylation-dependent manner, which indicates that malonylation/demalonylation is also involved in regulating innate immune response. Although Sirt5 was recently found to impair anti-viral innate immunity by desuccinylating MAVS 42, our data indicated that DDX3 was majorly malonylated and could be demalonylated by Sirt5, which also improve the function and mechanism of Sirt5. The different results may result from different VSV tilter and mice raising system, which will affect the expression and activation of Sirt5. We could not exclude the possibility that target protein specificity of Sirt5 under different activation signal. Further research of Sirt5 in anti-viral innate immunity needs to be conducted. Given that all Sirtuins are deacylases, whether the other Sirtuins also participate in regulating antiviral immune response needs further studies. Accordingly, the types and function of PTM, such as phosphorylation and acylation, of Sirt5 itself also needs to be further investigated.

DDX3 has been reported to promote IFN-β production and antiviral immune through multiple mechanisms. The C-terminus of DDX3 enhances IPS-1 function, while the N-terminus interacts with IKKε and TBK1 in a virus-dependent manner to promote *Ifnβ* transcription 33, 35, 43. Additionally, DDX3 directly binds to *Ifnβ* promoter and induces IFNβ transcription 35. Recent studies have found that DDX3 acts as an HIV-1 sensor that binds to abortive HIV-1 RNA and triggers type I interferon responses via TRAF3 recruitment to MAVS to suppress HIV-1 replication 34, 44. Moreover, DDX3 restricts HBV transcription and DEVN replication as an intrinsic host antiviral factor 32, 45. DDX3 belongs to the DExD/H box RNA helicase families that are important components of the innate immune system. Several RNA helicases have been reported to function as sensors or signal transducers of both microbial RNA and DNA, including RIG-I 46. Our study reveals DDX3 as a substrate of Sirt5 and demonstrates the mechanism of activation of DDX3. The finding that malonylation on lysine 66, 130 and 162 inhibits while acetylation on lysine 118 promotes its function on *Ifnβ* transcription not only indicates the different acylation regulates precisely DDX3 function, but also reveals there are other acetylases and deacetylases to regulate DDX3. However, whether DDX3 senses and directly binds to viral RNA as a pattern-recognition receptor and whether the demalonylation of DDX3 by Sirt5 affects this process needs to be explored further in the future. Our finding that DDX3 activation through also promotes STING signaling, which also provides novel cancer therapy targets 47, 48.

## Supplementary Material

Supplementary figures and tables.

## Abbreviations

IFN-I: Type I interferon
MAVS: Mitochondrial antiviral signaling protein
TBK1: Tank-binding kinase 1
IRF3: Interferon regulatory factor 3
ERK: Extracellular signal regulated kinase 1
JNK: c-Jun N-terminal kinase
MAPK: Mitogen-activated protein kinases
NF-κB: Nuclear factor-κB
VSV: Vesicular stomatitis virus
HSV: Herpes simplex virus
