# Supplementary material for: Demalonylation of DDX3 by Sirtuin 5 promotes antiviral innate immune responses
Source: Theranostics. 2021 May 24;11(15):7235–46. doi: 10.7150/thno.52934 (PMC8210596; doi:10.7150/thno.52934)
Supplement: Supplementary file 1 — Supplementary figures and tables. [file thnov11p7235s1.pdf]

Demalonylation of DDX3 by Sirtuin 5 promotes antiviral innate immune responses

Xingying He[1#], Tianliang Li[2#], Kewei Qin[3#], Shiyuan Luo[4,#], Zhenjie Li[1#],Qingqing Ji[1], Honghao Song[1], Huyang He[1], Hao Tang[5]*, Chaofeng Han[6]*, Hongjiao Li[7]*, Yan Luo[4]*

sFig. 1

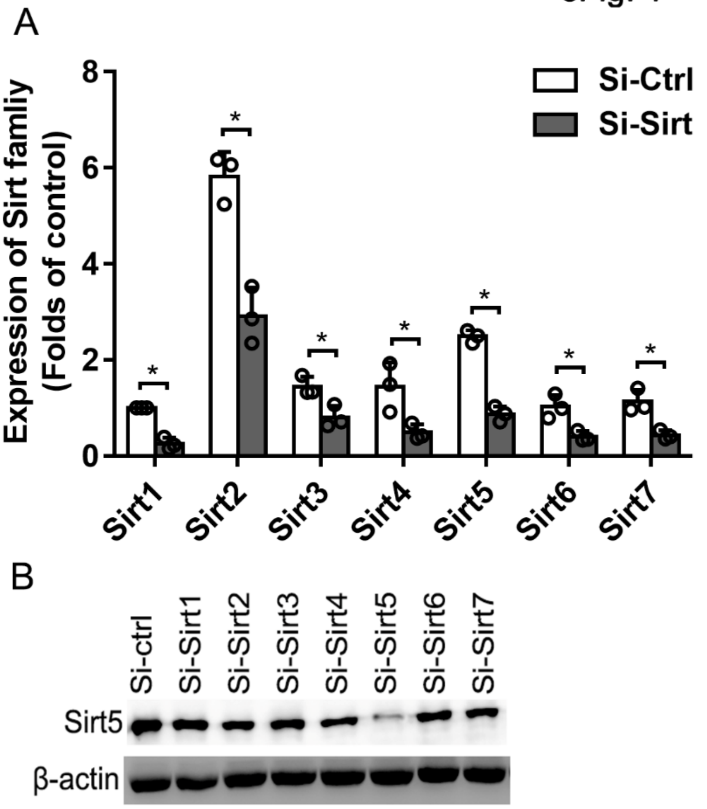

Supplementary Figure 1. (A) Q-PCR analysis of knockdown efficiency in BMDMs transfected with siRNA specific for Sirt1-7 for 48h. The results are normalized to *Gapdh* expression. (B) Immunoblot analysis of BMDMs transfected with indicated Si-RNA for 48 h and then infected with VSV for 8h with indicated antibodies. Data are presented as mean ± standard deviation (SD) of three independent experiments. (* $P<0.05$)

sFig. 2

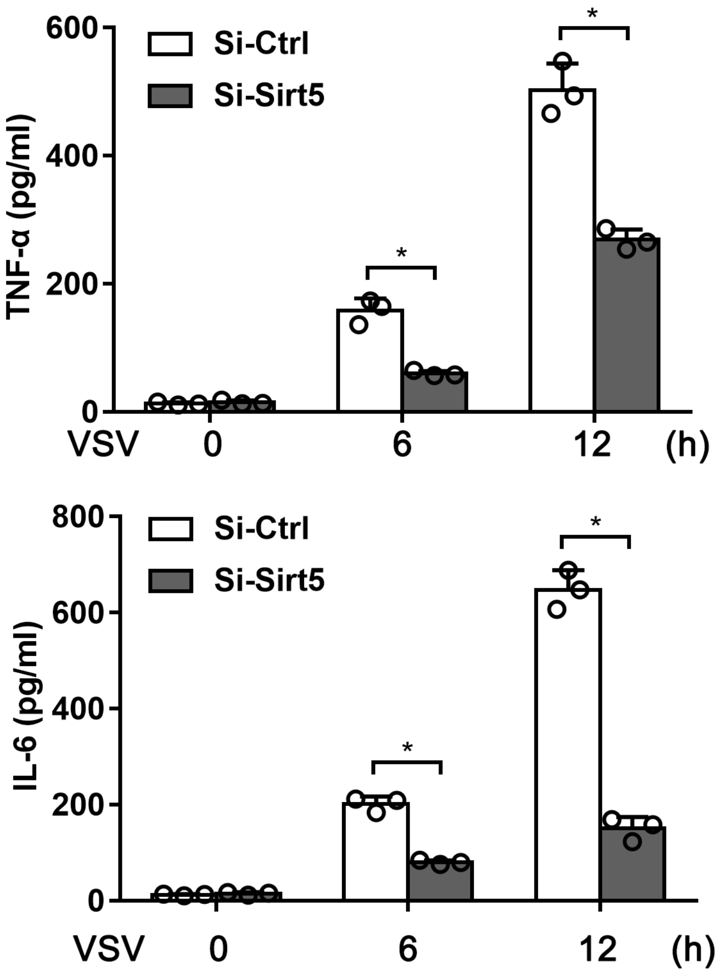

Supplementary Figure 2. ELISA of TNFα and IL-6 production in supernatants from BMDMs transfected with siRNA specific for Sirt5 for 48h and then infected with VSV for indicate time. Data are presented as mean ± standard deviation (SD) of three independent experiments. (* $P<0.05$)

sFig. 3

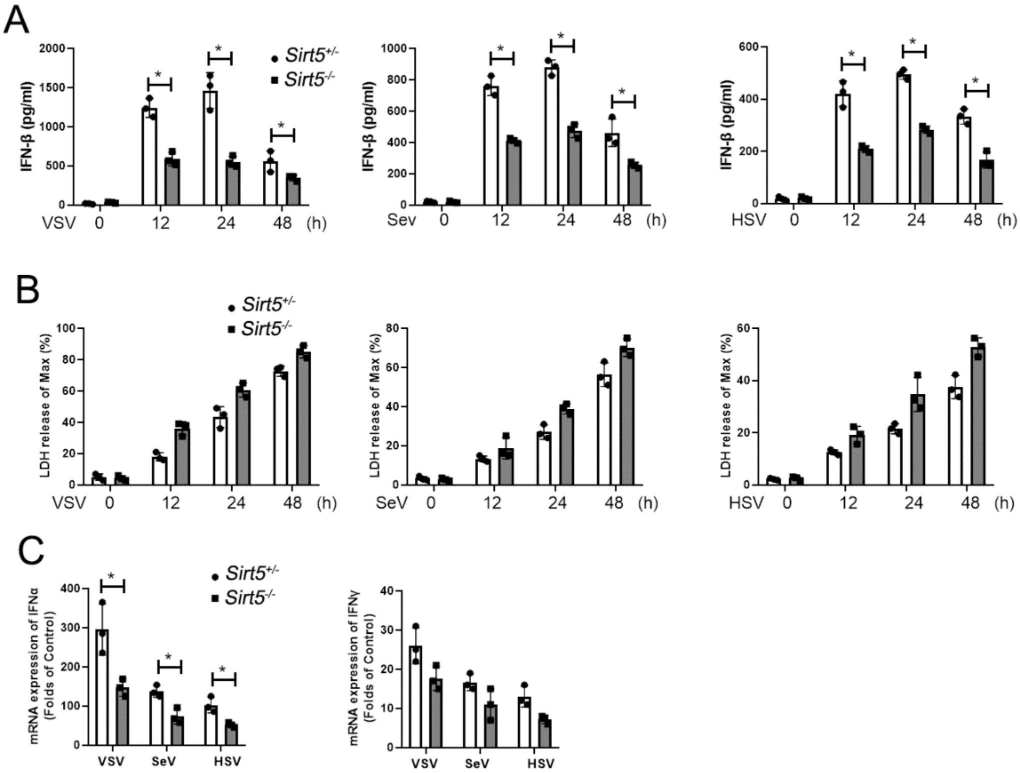

Supplementary Figure 3. (A), ELISA of IFN-β production in supernatants from *Sirt5*$^{+/-}$ or *Sir5*$^{-/-}$ BMDMs infected with VSV, SeV and HSV with indicated time. (B) Cytotoxicity was measured by subtracting LDH content in supernatants from *Sirt5*$^{+/-}$ or *Sir5*$^{-/-}$ BMDMs infected with indicated viruses compared to total LDH in untreated controls. (C) Q-PCR of IFN-α and IFN-γ mRNA expression in *Sirt5*$^{+/-}$ or *Sir5*$^{-/-}$ BMDMs infected with VSV, SeV and HSV for 8 hours. Data are presented as mean ± standard deviation (SD) of three independent experiments. (* P<0.05)

sFig. 4

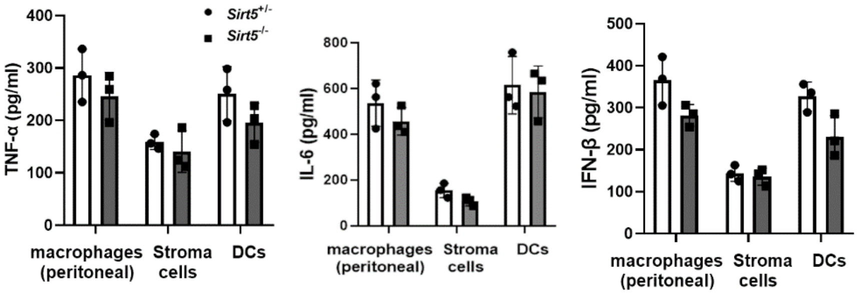

Supplementary Figure 4. ELISA of TNFα, IL-6 and IFN-β production in supernatants from *Sirt5*$^{+/-}$ or *Sir5*$^{-/-}$ peritoneal macrophages, bone marrow stroma cells and DCs stimulated with ssRNA40/Lyo Vec™ (1 µg/ml) for 12 hours. Data

are presented as mean ± standard deviation (SD) of three independent experiments.

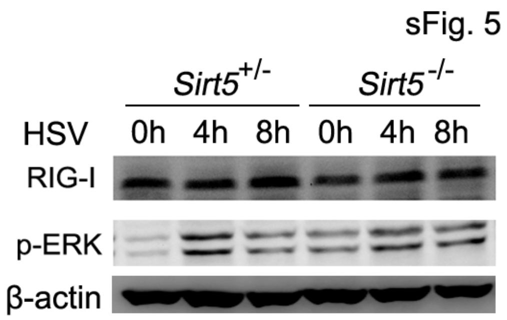

Supplementary Figure 5. Immunoblot analysis of *Sirt5*$^{+/-}$ or *Sir5*$^{-/-}$ BMDMs infected with HSV for indicated time and with indicated antibodies. Data are representative of three independent experiments.

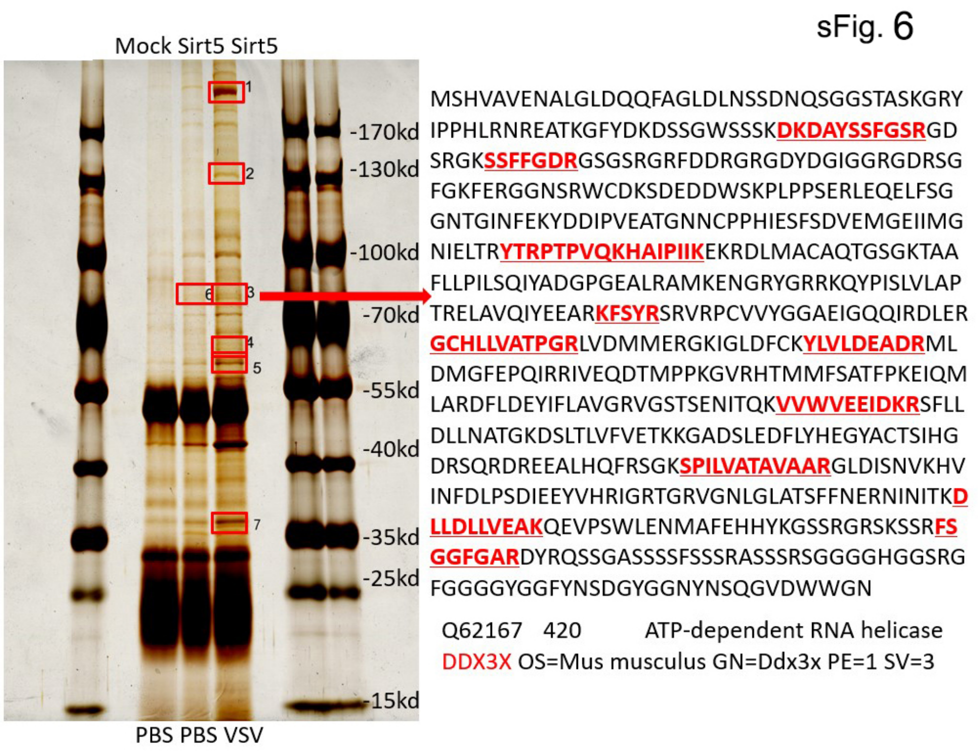

Supplementary Figure 6. Mass-Spectro analysis of Sirt5 interacting proteins. Raw264.7 cells overexpressed with Flag-tagged Sirt5 or empty vector were infected with VSV for 6h. The Flag-tagged SIRT5 was immunoprecipitated and with anti-Flag antibody and loaded on SDS-page. After sliver staining, the indicated bands were analyzed by Mass-Spectro and the peptides gained from band 3 were marked in red color in the sequence of DDX3.

supplementaryTable1.MASS-SPECTROdata

Require bo 0

Protein hit---

prot_hit_nu	prot_acc	prot_desc	prot_score	prot_mass	prot_matcl	prot_matcl
9	Q62167	ATP-deper	775	73455	38	20
9	Q62167	ATP-deper	775	73455	38	20
9	Q62167	ATP-deper	775	73455	38	20
9	Q62167	ATP-deper	775	73455	38	20
9	Q62167	ATP-deper	775	73455	38	20
